# Suicide Postvention Service Models and Guidelines 2014–2019: A Systematic Review

**DOI:** 10.3389/fpsyg.2019.02677

**Published:** 2019-11-29

**Authors:** Karl Andriessen, Karolina Krysinska, Kairi Kõlves, Nicola Reavley

**Affiliations:** ^1^Centre for Mental Health, School of Population and Global Health, The University of Melbourne, Carlton, VIC, Australia; ^2^Orygen, The National Centre of Excellence in Youth Mental Health, Parkville, VIC, Australia; ^3^WHO Collaborating Centre for Research and Training in Suicide Prevention, School of Applied Psychology, Australian Institute for Suicide Research and Prevention, Griffith University, Brisbane, QLD, Australia

**Keywords:** bereavement, guidelines, mental health, postvention, suicide, systematic review

## Abstract

**Background:** Suicide bereavement can have a lasting and devastating psychosocial impact on the bereaved individuals and communities. Many countries, such as Australia, have included postvention, i.e., concerted suicide bereavement support, in their suicide prevention policies. While little is known of the effectiveness of postvention, this review aimed to investigate what is known of the effects of postvention service delivery models and the components that may contribute to the effectiveness.

**Method:** Systematic review and quality assessment of peer reviewed literature (Medline, PsycINFO, Embase, EBM Reviews) and gray literature and guidelines published since 2014.

**Results:** Eight studies and 12 guidelines were included, with little evidence of effectiveness. Still, providing support according to the level of grief, involvement of trained volunteers/peers, and focusing the interventions on the grief, seem promising components of effective postvention.

**Conclusions:** Adopting a public health approach to postvention can allow to tailor the service delivery to needs of the bereaved individuals and to align postvention with suicide prevention programs.

## Introduction

### Rationale

Suicide is a major public and mental health problem in Australia. Over the last 10 years the country has witnessed a 33% increase of the annual number of suicides, from 2,341 in 2008 to 3,128 in 2017 (Australian Bureau of Statistics, 2018). The age-standardized suicide rate (per 100,000 persons) increased from 10.9 in 2008, to 12.6 in 2017, which is higher than the global age-standardized suicide rate of 10.5/100,000 persons (Australian Bureau of Statistics, 2018; World Health Organization, 2018). While the increasing suicide mortality has fueled calls for evidence-based suicide prevention, concern has also increased for the many bereaved family members, friends and community members (Department of Health, 2017). Indeed, experiencing bereavement by suicide can be a major stressor, increasing the risks of social, physical, and mental health problems, and suicidal behavior in the bereaved individuals (Pitman et al., 2014; Andriessen et al., 2017a). The impact of suicide on society can be far-reaching. Studies have shown that, on average, five immediate family members and up to 135 individuals can be exposed to the impact of an individual's suicide (Berman, 2011; Cerel et al., 2018). A recent meta-analysis determined that approximately one in 20 people (4.3%) are impacted by a suicide in any 1 year, and one in five (21.8%) during their lifetime (Andriessen et al., 2017b).

Grief is the natural reaction to the loss of a close person such as a family member or a friend (Stroebe et al., 2008). As with grief due to other causes, grief after suicide can include diverse psychological, physical, and behavioral responses to the death (Andriessen et al., 2017a). Feelings of sadness, yearning, guilt and anger, and physical reactions such as crying, are common grief reactions (Stroebe et al., 2008). People exposed to a suicide death can be affected to varying degrees. Those who were psychologically close to the person who has died are likely to be more strongly affected than those whose relationships were more distant. Cerel et al. (2014) proposed a theoretical continuum of suicide survivorship ranging from those who are merely exposed to a suicide without experiencing an impact on their life, to those who feel affected or distressed, to those who experience intense short or long-term grief reactions.

The course and duration of the grief process after a suicide death seem similar to grief processes after other causes (Sveen and Walby, 2008; Jordan and McIntosh, 2011). However, people bereaved by suicide may experience more shock or trauma related to the unexpected or violent nature of the death, and more feelings of abandonment, rejection, shame, and struggles with meaning-making and “why”-questions. They may also experience less social support compared to other forms of bereavement, which may be due both to limited help-seeking or sharing by the bereaved individuals and the inability of the social network to support them (Andriessen et al., 2017a; Pitman et al., 2017).

Suicide bereavement is a risk factor for complicated or prolonged grief (Mitchell et al., 2004). This is expressed through persisting characteristics of acute grief, such as intense longing and ruminative thoughts about the deceased, avoidance of situations related to the loss, and difficulty finding meaning in life (Zisook et al., 2014; Malgaroli et al., 2018; Mauro et al., 2019). Compared with the general population, people bereaved by suicide have a two- to three-fold risk of suicidal behavior, and psychiatric problems, such as depression, anxiety, posttraumatic stress disorder, and substance abuse (De Groot and Kollen, 2013). Having a personal or family history of mental health and/or suicidal behavior increases the risks of these problems (Pitman et al., 2014; Erlangsen and Pitman, 2017). People bereaved by suicide are also susceptible to physical illnesses, possibly due to the levels of stress or an unhealthy lifestyle (e.g., poor diet, smoking) (Erlangsen and Pitman, 2017; Erlangsen et al., 2017; Spillane et al., 2018).

Recent research has also started to shed light on the phenomenon of personal (or posttraumatic) growth in suicide bereavement (Levi-Belz et al., under review). This has been defined as the positive psychological changes experienced by an individual as the result of inner struggles after a traumatic experience (Tedeschi et al., 2018). While the research into positive personal transformations in the context of suicide bereavement is still new, it reveals that the aftermath of suicide is not always simply deleterious, and personal growth is possible.

In summary, loss by suicide can have serious and lasting psychosocial effects on the bereaved individuals and communities. Their needs are complex and variegated, necessitating a concerted provision of support.

### Policy Response

The Commonwealth and the state/territory suicide prevention policies and documents in Australia recognize the importance of postvention in the overall suicide prevention efforts and the involvement of the bereaved in shaping these actions. According to the Fifth National Mental Health and Suicide Prevention Plan (Department of Health, 2017), suicide prevention efforts call for a broad approach involving a range of sectors, and targeting various settings, populations, and risk groups. Postvention, i.e., an improved response to and caring for people affected by suicide, is an element of a systems-based approach informing the Fifth Plan (Department of Health, 2017), originally based on the World Health Organization's seminal Preventing suicide: A global imperative report (World Health Organization, 2014). The Fifth Plan promises that “there will be improved postvention support for carers, families and communities affected by suicide” (Department of Health, 2017, p. 25).

The voices of people bereaved by suicide have been included in the development of the Strategic Framework for Suicide Prevention in NSW 2018–2023 (Mental Health Commission of NSW, 2018). Postvention programs and services which are “co-designed, inclusive, coordinated and integrated” (Mental Health Commission of NSW, 2018, p. 11) are included under one of the five goals of the Framework, along with suicide prevention and intervention initiatives. The Framework's Priority Area 2 involves strengthening the community response to suicide and points out to the needs of communities to be able to respond to people bereaved by the death. People bereaved by suicide may be at increased risk of suicide themselves and require timely and effective support, such as grief counseling and advice on how to find relevant services. Promotion of “community-based postvention support, tools and resources for families and communities” (Mental Health Commission of NSW, 2018, p. 26) after a suicide is one of the important actions that require immediate attention of the NSW Government. Further, the NSW Framework recognizes the potential of professionalized suicide prevention peer workforce, comprising people bereaved by suicide, in reducing the number of suicides (Mental Health Commission of NSW, 2018).

### Postvention Services

It has long been recognized that people bereaved by suicide have diverse psychosocial and health needs (Shneidman, 1973) and effective postvention, i.e., suicide bereavement support, is seen as a major public and mental health challenge. Andriessen (2009, p. 43) defined postvention as: “those activities developed by, with, or for suicide survivors, in order to facilitate recovery after suicide, and to prevent adverse outcomes including suicidal behavior.”

Since the 1960 various forms of postvention services and support programs have been developed. These include group support, grief counseling, outreach by agencies, and online support (McIntosh et al., 2017). Some postvention programs are focused on specific settings, such as schools (Cox et al., 2016), workplaces (Spencer-Thomas and Stohlmann-Rainey, 2017), and faith communities (Krysinska et al., 2017), while other initiatives aim to provide support to the broader community (Andriessen et al., 2017c). Historically, most postvention services were initiated by the bereaved people themselves, followed by involvement of professionals (Farberow, 2001). Originally scarce, in recent years, progress has been made regarding the availability of postvention services both internationally and in Australia (https://postventionaustralia.org/finding-support/; http://www.supportaftersuicide.org.au/find-related-organizations) (McIntosh et al., 2017).

Suicide bereavement support groups are the most widely available postvention services. Frequently initiated by people bereaved by suicide, they are often based on the principles of sharing experiences and offering mutual assistance, thereby reducing distress and risk of mental and emotional problems (McIntosh, 2017). Support groups can be facilitated by survivors, mental health professionals, or a combination of both (McIntosh, 2017). While “open” groups are ongoing and accept new members, “closed” groups meet for a predetermined number of times with the same participants (Farberow, 2001; McIntosh, 2017).

Some people bereaved by suicide experience emotional (e.g., shame) or physical barriers (e.g., limited availability of services) to contacting a support group. Anticipating such barriers, some organizations have developed an outreach approach in which the service contacts the bereaved person after being notified of a suicide by the police or the coroner's office (McIntosh et al., 2017; Mowll et al., 2017). Such a pro-active approach has a potential to improve the collaboration between first-responders (e.g., police) and suicide bereavement services. It may also decrease the time elapsed between the suicide and the start of support received, though the effect of the outreach approach on the grief process remains unknown (Cerel and Campbell, 2008; Comans et al., 2013).

The Internet has become a major source of suicide bereavement information and support provided via websites, discussion forums, social media, and online memorials (Krysinska and Andriessen, 2017). Compared to face-to-face support, users of online services may have more control over the process and content of the interventions, which may be particularly important for people who feel stigmatized or are reluctant to access other forms of support. However, dropout rates tend to be higher online relative to interventions provided face-to-face (Karyotaki et al., 2015). As in face-to-face support groups, participants in online forums or groups can share personal stories, which may help to normalize their grief experiences (Krysinska and Andriessen, 2017). They can also find and provide empathy, mutual support and hope through the exchange of resources or advice (Schotanus-Dijkstra et al., 2014).

In some countries, support groups and/or other suicide bereavement services have created national networks or associations, such as the Suicide Loss Division of the American Association of Suicidology in the USA (https://www.suicidology.org/), the Support After Suicide Partnership in the UK (http://supportaftersuicide.org.uk/) (Lascelles et al., 2017), and Postvention Australia (https://postventionaustralia.org/) (Ceramidas et al., 2017). There is also increasing international collaboration, for example, through the Special Interest Group on Suicide Bereavement and Postvention of the International Association for Suicide Prevention (https://www.iasp.info/). Some of these organizations have developed guidelines on how to facilitate a support group (World Health Organization and International Association for Suicide Prevention, 2008), or national guidelines for suicide bereavement support (Jordan, 2017).

Overall, there is a tension between the need for psychosocial services for people bereaved by suicide (Sanford et al., 2016; Pitman et al., 2018) and what is known about their effectiveness (McDaid et al., 2008; Szumilas and Kutcher, 2011; Linde et al., 2017). Indeed, despite the devastating and lasting effects a suicide can have on people bereaved by suicide, and the number of people affected, little is known about what services and supports are effective. Postvention has been recognized as an important suicide prevention strategy in Australia and worldwide (World Health Organization, 2014; Department of Health, 2017). Still, most research has been focused on the characteristics of suicide bereavement rather than on effectiveness of interventions (Andriessen, 2014; Andriessen et al., 2017d; Maple et al., 2018). Our recent systematic review of grief and psychosocial interventions for people bereaved through suicide, which included only controlled studies, found mixed evidence of effectiveness of interventions (Andriessen et al., 2019). Clearly, further examination of the quality of postvention research, levels of evidence, and potentially effective postvention components, is needed.

### Research Questions

This review was designed to answer the following two research questions.

#### Question 1

Which suicide postvention service models have been shown to be effective to reduce distress in family, friends and communities following a suicide?

#### Question 2

From the models identified in Question 1, what components of suicide postvention services have been determined to contribute to effectiveness?

We defined “suicide postvention service model” as a “coordinated approach to providing support to people impacted by the death of a family member, friend or person in a network (such as a school, nursing home, workplace, etc.) through suicide.” As we were interested in current service models, we focused the review on peer reviewed literature, gray literature and guidelines published over the last 5 years.

## Methods

### Peer Review Literature

#### Search Strategy

We developed the search strategy of this review based on experiences of our team in conducting rapid and systematic reviews (e.g., Krysinska et al., 2018; Andriessen et al., 2019). In line with the PRISMA guidelines (http://www.prisma-statement.org/) (Moher et al., 2009), we conducted systematic searches of the following databases: Medline, PsycINFO, Embase, and EBM Reviews. All databases were accessed through Ovid. The search string in Medline comprised a combination of MeSH and keywords: (bereavement/ OR bereavement.mp OR grief/OR grief.mp OR mourning.mp) AND (family/OR friends/ OR friends.mp OR acquaintance.mp OR students/OR student.mp OR schools/OR school.mp OR survivor.mp OR suicide survivor.mp) AND (counseling/OR counseling.mp OR intervention.mp OR postvention.mp OR psychotherapy/OR psychotherapy.mp OR support group.mp OR self-help groups/ OR social media/OR social media.mp OR internet/OR internet.mp) AND (suicide/OR suicide.mp OR suicide cluster.mp). We applied the same search string in the other databases using subject headings and keywords.

**Figure 1:**
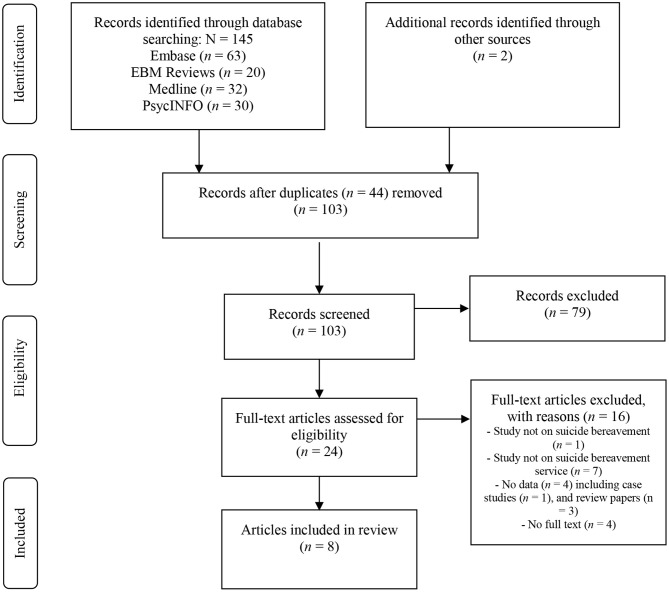
PRISMA flow diagram.

The search was undertaken in April 2019, was not limited by language, and comprised the years 2014 to 2019. Two researchers (KA, KKr) independently assessed titles and abstracts for eligibility. We resolved any disagreement through discussion. Potentially relevant studies were examined against the inclusion/exclusion criteria. The references of retrieved papers and existing reviews were hand searched to identify additional studies. Figure 1 presents the search and selection process.

#### Inclusion and Exclusion Criteria

Original studies published in peer-reviewed journals were included if: (1) the study population consisted of people bereaved by suicide, (2) the study applied quantitative, qualitative or mixed-methods, and (3) the study reported data regarding effects of interventions or service delivery to the study population. The review excluded studies: (1) not on suicide bereavement, (2) not providing original data (such as review papers), (3) not reporting on suicide postvention services, and (4) full-text not available (i.e., conference abstract).

#### Data Extraction

Two researchers (KA, KKr) independently extracted the following data from the selected studies: study reference including author, year and location (country), study design, assessments, sample size, participants' age and sex distribution, participants' relationship to the deceased and time since the bereavement, type (individual, family, group), characteristics and setting of the intervention, outcome measures and names of the instruments used, main outcomes of the study, and study limitations. Any disagreement was resolved through discussion. The data extraction informed the synthesis and report of the data.

#### Quality Assessment

We assessed the quality of the included studies using two instruments: the (National Health and Medical Research Council, 2009) NHMRC Levels of Evidence, and the Quality Assessment Tool for Quantitative Studies (Effective Public Health Practice Project, 1998).

The NHMRC Levels of Evidence comprises six levels of evidence based on the design of the study (Supplementary Table 1) (National Health and Medical Research Council, 2009). Systematic reviews of randomized controlled trials (RCTs) are considered the highest level of evidence (Level I). Case series, with post-test or pre- and post-test outcomes are at the bottom of the evidence hierarchy (Level IV). The NHMRC instrument also requires a summary of the body of evidence of five components: evidence-base (e.g., number and quality of the studies), consistency of findings across studies, clinical impact, generalizability of findings, and applicability in the Australian or local context (Supplementary Table 2). Two researchers (NR, KA) independently assessed the levels of evidence, and settled any disagreement through discussion.

The Quality Assessment Tool for Quantitative Studies (Effective Public Health Practice Project, 1998) comprises six components (selection bias, study design, confounders, blinding, data collection methods, and withdrawals and dropouts) which are scored as “strong,” “moderate” or “weak.” Complying with the instructions of the instrument, the total rating of a study was “strong” if none of its components were rated “weak.” We rated a study as “moderate” if only one of its components was rated “weak,” and rated a study as “weak” if two or more of its components were rated as “weak” (Effective Public Health Practice Project, 1998). In addition, the instrument assesses the integrity of the intervention and analyses (e.g., analysis by intention to treat status). Two researchers (KKr, KA) independently assessed the quality of the included studies and settled any disagreement through discussion.

### Gray Literature and Guidelines

#### Search Strategy

Guidelines are usually defined as information on how something should be done (AGREE Next Steps Consortium, 2017). More specifically, clinical practice guidelines are defined as “systematically developed statements to assist practitioner and patient decisions about appropriate health care for specific clinical circumstances” (AGREE Next Steps Consortium, 2017). As such, guidelines differ from general advice or a list of resources.

We developed a search strategy based on previous experiences of our team (Krysinska and Andriessen, 2010; Krysinska et al., 2018) and indications from the literature (Eysenbach and Köhler, 2002; Morahan-Martin, 2004; Jansen and Spink, 2006). The searches were conducted in April 2019 in Google Chrome. For each search term we opened a new page using Guest Mode to avoid that browser history affected the results. We used the following search terms: “suicide bereavement support,” “suicide loss support,” “suicide survivor support,” “effective suicide bereavement support,” “effective suicide loss support,” “effective suicide survivor support,” “suicide bereavement service,” “suicide loss service,” “suicide survivor service,” “effective suicide bereavement service,” “effective suicide loss service,” “effective suicide survivor service,” “postvention support,” “postvention service,” “effective postvention support,” “effective postvention service,” “support after suicide,” “help after suicide,” “effective support after suicide,” “effective help after suicide,” “postvention guidelines,” “suicide loss guidelines,” and “suicide bereavement guidelines.”

Research regarding how people search for health-related information on the Internet shows that most people only access links provided on the first page (Eysenbach and Köhler, 2002; Morahan-Martin, 2004), and the proportion of people viewing the first page only, has increased over the years (Jansen and Spink, 2006). To capture the research on services and guidelines that are readily available to the public, and to be thorough in the gray literature searches, we retained the results of the first two pages per search term. As such, the searches aimed to identify as many research publications and best-practice guidelines as possible, while confining the leads to a manageable number.

**Figure 2:**
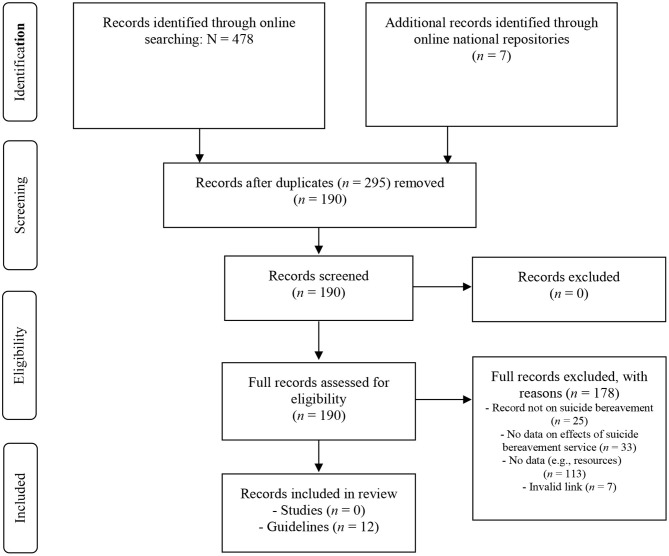
PRISMA flow diagram: gray literature.

In addition to the Google Chrome searches, we consulted the following national repositories of suicide prevention resources in English-speaking countries: The Suicide Prevention Hub, Australia (https://suicidepreventionhub.org.au/), National Office for Suicide Prevention, Ireland (https://www.hse.ie/eng/services/list/4/mental-health-services/nosp/), Support After Suicide Partnership, UK (http://supportaftersuicide.org.uk/), Suicide Prevention Resource Center, USA (https://www.sprc.org/resources-programs), Centre for Suicide Prevention, Canada (https://www.suicideinfo.ca/), Mental Health Foundation, New Zealand (https://www.mentalhealth.org.nz/). Two researchers (KKo, KA) independently assessed the leads for eligibility. Any disagreement was resolved through discussion and/or involvement of a third researcher (KKr). Figure 2 summarizes the search and selection process for the gray literature.

#### Inclusion and Exclusion Criteria

We adapted the inclusion and exclusion criteria of the peer-reviewed literature (above) to the search of the gray literature. Studies in webpages were included if: (1) they reported on a study population consisting of people bereaved by suicide, (2) the study applied quantitative, qualitative or mixed-methods, and (3) reported data regarding effects of interventions or service delivery to the study population. The review excluded studies: (1) not on suicide bereavement, (2) not providing original data of effects of interventions (e.g., presenting case histories or description of services), (3) not reporting on suicide postvention services (e.g., webpages limited to written resources, links, or referral addresses), and (4) invalid links.

The gray literature review included guidelines published since 2014 if: (1) they self-identified as “guidelines” and/or (2) comprised a structured set of statements on how an organization or a service can provide help to individuals bereaved by suicide. The review excluded documents (1) comprising a collection of resources, (2) providing general advice on how to support a person bereaved by suicide or self-care information for the bereaved.

#### Data Extraction

The gray literature search did not identify any studies not previously identified through the peer-review literature searches. Based on the criteria provided in the “Appraisal of Guidelines for Research and Evaluation II” instrument (AGREE Next Steps Consortium, 2017), two researchers (KA, KKr) independently extracted the following data from guidelines included in the review: reference including title, author, year and location (country), target users, target population, whether objectives and methods of development were described, if target users were involved in the development, whether the evidence-base of the guidelines and the theoretical model of postvention were described, and whether key recommendations or sample material, such as templates, were included. We resolved any disagreement through discussion. The data extraction informed the synthesis and report of the data.

## Results

### Study Characteristics

Eight papers published since 2014 met the inclusion criteria and were included in the review (Table 1). Two studies were conducted in Australia (Visser et al., 2014; Peters et al., 2015), two in the USA (Supiano et al., 2017; Zisook et al., 2018), two in Belgium (Wittouck et al., 2014) (including one also conducted in the Netherlands, Kramer et al., 2015), and one in Korea (Cha et al., 2018) and Italy (Scocco et al., 2019), each. There were two RCTs (Wittouck et al., 2014; Zisook et al., 2018), two pre- and post-designs without control group (Kramer et al., 2015; Scocco et al., 2019), two prospective designs without control groups (Supiano et al., 2017; Cha et al., 2018), and two retrospective descriptive, cross-sectional studies (Visser et al., 2014; Peters et al., 2015).

**Table 1:** Summary of included studies.

**Study reference,** **location**	**Study design,** **assessment**	**Level of evidence (NHMRC grade)**	**Sample** **intervention/control** ***N* = …** **Age: M (SD) or range** **Sex: F/M: n/n (%/%)**	**Intervention,** **setting**	**Outcome measures**	**Main outcomes**	**Limitations**
Cha et al. (2018) Korea	Prospective cohort study Assessment: -Baseline: 1 week after suicide -Follow-up at 5 months	III-3	*N* = 956 F/M: 506/450 (53%/47%) Trauma-symptom group (CROPS ≥ 19) *N* = 83 Age: *M* = 17.1 (*SD* 0.8) F/M: 57/26 (69%/31%) Non-trauma group (CROPS <19) *N* = 873 Age: *M* = 16.9 (*SD* 0.8) F/M: 449/424 (51%/49%)	A school-based crisis intervention program conducted 1 week after a peer suicide. Program included screenings, educational sessions, and further interview with psychiatric specialists for the trauma-symptom group. Setting: School	Posttraumatic stress symptoms: Child Report of Posttraumatic Symptoms (CROPS) The University of California at Los Angeles posttraumatic stress disorder (PTSD) reaction index (UCLA-PTSD-RI) Anxiety symptoms: Korean-Beck Anxiety Inventory (K-BAI) Depressive symptoms: Korean-Beck Depression Inventory-II (K-BDI-II) Complicated grief: Inventory of Complicated Grief (ICG)	Significant differences in CROPS, UCLA-PTSD-RI, K-BAI, K-BDI-II, and ICG scores between baseline and follow-up in both groups. Scores of the “trauma” group dropped more compared to the non-trauma group. At follow-up 2.9% of students were in the “trauma” group vs. 8.6% at baseline. A higher proportion of female students showed posttraumatic stress symptoms than male students.	Timing of follow-up determined by school circumstances Various psychosocial factors not examined, such as level of psychological closeness between the deceased and the students, social support, family functioning, or pre-existing psychopathology No unexposed control group
Kramer et al. (2015) Belgium, and The Netherlands	Pre-/post study Mixed methods: self-reported measures and interviews Assessment: -Baseline -Follow-up at 6 and 12 months -Interviews with selected sample after 12 months	IV	*N* = 270 Age: *M* = 42.9 (*SD* 12.4) F/M: 238/32 (87%/13%) Interview subgroup: *n* = 29 Age: *M* = 45.3 (*SD* 10.8) F/M: 26/3 (90%/10%)	Two government-funded web-based peer support forums for the bereaved by suicide. Site visitors can read and/or post messages about a specific topic. The two forums were similar in terms of layout, structure, and most of the predefined sub-forums. Setting: Online	Well-being: WHO-Five Well-being Index (WHO-5) Symptoms of depression: Center for Epidemiological Studies Depression Scale (CES-D) Complicated grief: Inventory of Traumatic Grief (ITG) Suicide risk: subscale of the MINI-International Neuropsychiatric Interview (MINI-Plus) Semi-structured interview about experiences with forum	Significant improvement in well-being and depressive symptoms (both *p* < 0.001). Small to medium pre-post effect sizes for well-being (6 months: *d* = 0.24, 12 months: *d* = 0.36), and small for depressive symptoms (6 months: *d* = 0.18, 12 months: *d* = 0.28). No change in grief symptoms (*p =* 0.08, 6 months: *d* = 0.05, 12 months: *d* = 0.12). No change in suicide risk (baseline: 20.8%. 12 months: 17.2%) Main reasons for visiting online fora: sharing with peers, finding recognition	Sample: online help-seeking, self-selected, mostly female Self-report measures subject to recollection bias High drop-out rate (43%) Dutch forum was launched 1 month before recruitment started, was not yet at its full capacity No control group
Peters et al. (2015) Australia	Retrospective study Mixed-methods: self-reported measures (online or hard copy) and interviews Assessment: shortly after intervention	IV	*N* = 82 Age: 75% over age 45 F/M: 75/7 (91%/9%) Interview subgroup: *n* = 30	The Lifekeeper Memory Quilt Project, implemented by the Suicide Prevention-Bereavement Support Services of the Salvation Army in 2008 to provide support for the bereaved by suicide and to create greater public awareness of suicide. Setting: Community-based	Participants' Evaluation of Quilt (PEQ-16): 16-item scale developed for the study to measure participant satisfaction Semi-structured interview about participants' experiences with project	High participant satisfaction (M 69.6; SD 9.1) According to 48%, 1 year after the loss was the best time for participating Approx. 92% rated the Quilt project as helpful or extremely helpful Qualitative analysis of the interviews found four themes: healing, creating opportunity for dialogue, reclaiming the real person, and raising public awareness.	Sample: mostly female, self-selected (55% response rate) People who participate in Quilt projects not necessarily representative Grief was not assessed Descriptive study No control group
Scocco et al. (2019) Italy	Pre-/post study Assessment: -Baseline: 4–6 days before intervention -Post: 4–6 days after	IV	*N* = 61 Age: *M* = 49.5 (*SD* 11.0) F/M: 49/12 (80%/20%)	A support program of mindfulness-based residential weekend retreats, including emotion- and grief-oriented exercises Setting: Residential, group	Mindfulness experiences: Five-Facet Mindfulness Questionnaire (FFMQ) Self-Compassion Scale (SCS) Dimensions of affect: Profile of Mood States (POMS)	Significant improvement over time in almost all dimensions of the POMS (mood states). No change in the dimensions of the SCS and FFMQ Compared with first-time participants, the multiple-participation group showed significant improvements over time on the Self-kindness subscale of the SCS and Non-judging subscale of the FFMQ	Sample: mostly female, help-seeking, self-selected participants Preferable, participants had attended self-help group/ counseling Unclear if observed effects were related to intervention or group effects Grief was not assessed No follow-up data No control group
Supiano et al. (2017) USA	Prospective, observational study Analysis of the process of individual participant change in three complicated grief therapy groups	IV	*N* = 21 Age: *M* = 53 (range 34–73) F/M: 15/6 (71%/29%)	Complicated grief group therapy (CGGT): a multimodal, manualized group psychotherapy, with 2-h sessions over 16 weeks Setting: Clinical, group	Meaning reconstruction in grief: Meaning of Loss Codebook (MLC) Grief and Meaning Reconstruction Inventory (GMRI)	Therapy facilitated resolution of complicated grief symptoms and integrated memory of the deceased The MLC codes captured most of the statements of participants, helped articulating the therapeutic process, and showed that CGGT facilitated grief. Some participants continued to experience physical distress, depression or anxiety, even with improved self-care.	Sample: small and mostly female Sample limited to people bereaved by suicide with complicated grief Findings may only be generalizable to persons seeking intensive psychotherapy No control group
Visser et al. (2014) Australia	Retrospective cross-sectional study Assessment: after intervention (unspecified)	III-3	Intervention: *N* = 90 Age: *M* = 45.7 (*SD* 15.8) F/M: 73/17 (82%/18%) Control: *N* = 360 Age: *M* = 40.1 (*SD* 13.4) F/M: 311/49 (88%/11%)	Face-to-face outreach and telephone support provided by a professional crisis response team. The service then develops a customized plan, referring clients to other community services matched to their needs. The service is provided only to people who request it. Setting: Community-based	Quality of life: EQ-5D^TM^ ICECAP index of capability Psychological distress: Kessler Psychological Distress Scale (K6) Suicidality: Suicidal Behaviors Questionnaire-Revised (SBQ-R) Work performance: World Health Organization Health and Work Performance Questionnaire (HPQ) Health care usage questions	Standby clients scored better on levels of suicidality (*p* = 0.006) No significant differences on other scales or health care usage	Sample: self-selected, mostly female Low response rate of clients (23%) Significant sociodemographic differences between the two groups Grief was not assessed Observational design, no control of confounding variables such as age of bereaved, time since death, and other treatments sought by participants
Wittouck et al. (2014) Belgium	Cluster RCT Assessment: Baseline 8-months after study entrance	II	Intervention: *N* = 47 Age: *M* = 49.3 (*SD* 13.8) F/M: 38/9 (81%/19%) Control/No treatment: *N* = 36 Age: *M* = 47.6 (*SD* 12.8) F/M: 25/11 (69%/31%)	Cognitive-behavioral therapy-based psychoeducational intervention, facilitated by clinical psychologists at participants' home 2 h sessions, 4 sessions, frequency not reported Setting: Clinical, group/family	Complicated grief: Inventory of Traumatic Grief, Dutch version (ITG) Depressive symptoms: Beck Depression Inventory (BDI-II-NL) Hopelessness: Beck Hopelessness Scale (BHS) Secondary outcomes: -Grief Cognitions Questionnaire (CGQ) -Utrecht Coping List (UCL)	No significant effect on the development of complicated grief reactions, depression, and suicide risk factors Secondary outcomes: Decrease in intensity of grief, depression, passive coping style, social support seeking and behavioral expression of negative feelings in intervention group only (all *p* < 0.05)	Sample: small, mostly female sample, possibly subject to selection bias Findings may only be generalizable to bereaved persons at-risk of complicated grief and/or seeking psychotherapy
Zisook et al. (2018) USA	RCT Assessment: -Baseline -Monthly -At week 20	II	Total: *N* = 395 -Suicide bereaved (SB): *n* = 58 -Accident/homicide (AH): *n* = 74 -Natural causes (NC): *n* = 263 Randomized in 4 groups: medication, placebo, CGT + medication, CGT + placebo Age: SB: *M* = 47.2 (*SD* 14.1) AH: *M* = 51.6 (*SD* 14.8) NC: *M* = 54.6 (S*D* 14.2) F/M: SB: 48/10 (82%/17%) AH: 56/18 (76%/24%) NC: 204/59 (78%/22%)	Manual-based structured Complicated Grief Therapy (CGT), facilitated by social workers, psychiatrists, psychologists Antidepressant medication (citalopram) with individual follow-up CGT: 16 sessions over 20 weeks Medication: 12-week with 2–4 weekly visits until week 20 Setting: Clinical, individual	Psychiatric symptoms: Structured Clinical Interview for DSM-IV-TR Axis 1 (SCID-1) Complicated grief: Complicated Grief Clinical Global Impressions Scale-Improvement (CG-CGI-I) Inventory of Complicated Grief (ICG) Structured Clinical Interview for Complicated Grief (SCI-CG) Grief-Related Avoidance Questionnaire (GRAQ) Suicidality: Columbia Suicide Severity Rating Scale-Revisited (C-SSRS-R) Impaired relationships: Work and Social Adjustment Scale (WSAS) Cognitions: Typical Beliefs Questionnaire (TBQ)	CGT was effective in all bereaved groups regarding CG symptom severity, suicidal ideation, grief-related functional impairment, avoidance and maladaptive beliefs. Lower improvement on clinician-rated CG-CGI-I in SB vs. AH and NC groups (*p* < 0.5) CGT seemed acceptable treatment in all groups Low acceptability of medication-only treatment	Sample: Underpowered to examine cause of death as a moderator and other possible interactions, for example related to socio-demographic variables High dropout rate in medication only subgroup Heterogeneity within cause of death subgroups No no-treatment control group

Seven studies (Visser et al., 2014; Wittouck et al., 2014; Kramer et al., 2015; Peters et al., 2015; Supiano et al., 2017; Zisook et al., 2018; Scocco et al., 2019) focused on adult populations, and one on young people (high school students) (Cha et al., 2018). While some studies (e.g., Supiano et al., 2017; Zisook et al., 2018) included older adults, no study specifically focused on them. Apart from the study of Cha et al. (2018), female participants outnumbered male participants, with the proportion of female participants ranging from 80 to 91%. The study populations consisted mainly of first-degree family members (Visser et al., 2014; Wittouck et al., 2014; Kramer et al., 2015; Peters et al., 2015; Zisook et al., 2018; Scocco et al., 2019), though most studies also included other relatives and/or non-relatives (Visser et al., 2014; Wittouck et al., 2014; Kramer et al., 2015; Cha et al., 2018; Zisook et al., 2018; Scocco et al., 2019). Time since loss in study participants varied considerably between studies, ranging from 1 week (Cha et al., 2018) to between 3 months and 30 years (Scocco et al., 2019). Reported mean time since loss ranged from *M* = 9.8 months (*SD* 5.7) (Wittouck et al., 2014) to *M* = 5.96 years (*SD* 3.7) (Peters et al., 2015).

The interventions were conducted in a variety of settings: clinical (Wittouck et al., 2014; Supiano et al., 2017; Zisook et al., 2018), community-based (Visser et al., 2014; Peters et al., 2015), residential (Scocco et al., 2019), school (Cha et al., 2018), and online (Kramer et al., 2015). Three studies involved a group intervention (Wittouck et al., 2014; Peters et al., 2015; Supiano et al., 2017), three studies an individual intervention (Visser et al., 2014; Kramer et al., 2015; Zisook et al., 2018), and two studies a combination of group and individual interventions (Cha et al., 2018; Scocco et al., 2019). Two interventions were described as manualized (Supiano et al., 2017; Zisook et al., 2018). Three interventions targeted individuals early in the grief process (Visser et al., 2014; Wittouck et al., 2014; Cha et al., 2018). Duration of intervention and the timing of participant assessment varied considerably between studies, ranging from assessment shortly after the intervention (e.g., Peters et al., 2015; Scocco et al., 2019) to assessment at 12-months follow-up (Kramer et al., 2015).

Studies differed regarding outcomes measured and instruments used. Most studies applied mental health measures, three studies (Visser et al., 2014; Kramer et al., 2015; Zisook et al., 2018) measured suicidality, and three studies did not assess grief (Visser et al., 2014; Peters et al., 2015; Scocco et al., 2019). No single measure was used in more than one study.

### Study Quality Assessment

Tables 2, 3 summarize the rating of the reviewed studies according to the NHMRC levels of evidence (National Health and Medical Research Council, 2009). There were two level II studies, two level III-3 studies, and four level IV studies (Table 2). Looking at the five components in detail, three were rated as “poor” (evidence-base, consistency, and clinical impact), and two were rated as “satisfactory” (generalizability and applicability) (Table 3).

**Table 2:** NHMRC levels of evidence.

**Study**	**NHMRC level of evidence**
Cha et al. (2018)	III-3
Kramer et al. (2015)	IV
Peters et al. (2015)	IV
Scocco et al. (2019)	IV
Supiano et al. (2017)	IV
Visser et al. (2014)	III-3
Wittouck et al. (2014)	II
Zisook et al. (2018)	II

**Table 3:** NHMRC matrix to summarize the evidence base.

**Component**	**Rating**
Evidence base	D (Poor)
Consistency	D (Poor)
Clinical impact	D (Poor)
Generalizability	C (Satisfactory)
Applicability	C (Satisfactory)

Table 4 summarizes the study quality according to the six components of the Quality Assessment Tool for Quantitative Studies (Effective Public Health Practice Project, 1998). The overall study quality was weak. One study received a rating of “strong” on four components (Wittouck et al., 2014), one study on three components (Zisook et al., 2018), and one study on two components (Supiano et al., 2017). The other studies were rated “strong” on only one component (Visser et al., 2014; Kramer et al., 2015; Peters et al., 2015; Cha et al., 2018; Scocco et al., 2019). Selection bias, blinding, and withdrawals and dropouts were the weakest components across studies. Two studies used randomized designs (Wittouck et al., 2014; Zisook et al., 2018); however, no studies reported the use of an intention-to-treat analysis. All studies appeared to have used valid and reliable measures. However, it is unknown if studies measured consistency of intervention (except for Supiano et al., 2017 and Zisook et al., 2018) and controlled for effects of other treatments (for example, by a family doctor) which participants might have been receiving.

**Table 4:** Summary of study quality.

**Quality criteria**	**Cha et al. (2018)**	**Kramer et al. (2015)**	**Peters et al. (2015)**	**Scocco et al. (2019)**	**Supiano et al. (2017)**	**Visser et al. (2014)**	**Wittouck et al. (2014)**	**Zisook et al. (2018)**
**A. Selection bias**								
Representativeness	Somewhat likely	Not likely	Not likely	Not likely	Not likely	Not likely	Not likely	Not likely
Percentage agreed	Can't tell	Can't tell	<60%	Can't tell	Can't tell	<60%	80–100%	Can't tell
Rating	Moderate	Weak	Weak	Weak	Weak	Weak	Weak	Weak
**B. Study design**								
Study design type	Cohort	Cohort	Other	Cohort	Other	Other	RCT	RCT
Described as randomized?	No	No	No	No	N.a.	No	Yes	Yes
Method of randomization described?	N.a.	N.a.	N.a.	N.a.	N.a.	N.a.	Yes	Yes
Method appropriate?	N.a.	N.a.	N.a.	N.a.	N.a.	N.a.	Yes	Yes
Rating	Moderate	Moderate	Weak	Moderate	Weak	Weak	Strong	Strong
**C. Confounders**								
Pre-intervention differences?	Yes	N.a.	N.a.	N.a.	N.a.	Yes	Yes	Yes
Percentage confounders controlled for	<60% (few or none)	N.a.	N.a.	N.a.	N.a.	<60% (few or none)	80–100%	<60% (few or none)
Rating	Weak	N.a.	N.a.	N.a.	N.a.	Weak	Strong	Weak
**D. Blinding**								
Outcome assessors were blinded?	No	No	No	No	Can't tell	No	No	Yes
Participants were blinded?	Can't tell	Can't tell	Can't tell	Can't tell	Can't tell	Can't tell	Can't tell	Yes
Rating	Weak	Weak	Weak	Weak	Weak	Weak	Weak	Strong
**E. Data collection methods**								
Valid measures?	Yes	Yes	Yes	Yes	Yes	Yes	Yes	Yes
Reliable measures?	Yes	Yes	Yes	Yes	Yes	Yes	Yes	Yes
Rating	Strong	Strong	Strong	Strong	Strong	Strong	Strong	Strong
**F. Withdrawals and dropouts**								
Numbers and reasons reported per group?	No	No	N.a.	No	Yes	N.a.	Yes	No
Percentage completing study?	80–100%	<60%	N.a.	80–100%	80–100%	N.a.	80–100%	<60%
Rating	Weak	Weak	N.a.	Weak	Strong	N.a.	Strong	Weak
Total A-F:	WEAK	WEAK	WEAK	WEAK	WEAK	WEAK	WEAK	WEAK
Number of “strong” ratings	1/6	1/6	1/6	1/6	2/6	1/6	4/6	3/6
**G. Intervention integrity**								
Percentage participants received intervention?	80–100%	80–100%	80–100%	80–100%	80–100%	80–100%	80–100%	60–79%
Intervention consistency measured?	Can't tell	Can't tell	Can't tell	Can't tell	Yes	Can' tell	Can't tell	Yes
Confounding unintended intervention?	Can't tell	Can't tell	Can't tell	Can't tell	Can't tell	Can't tell	Can't tell	Can't tell
**H. Analyses**								
Unit of allocation	Individual	Individual	Individual	Individual	Individual	Individual	Individual	Individual
Unit of analysis	Individual	Individual	Individual	Individual	Individual	Individual	Individual	Individual
Appropriate statistical methods?	Yes	Yes	Yes	Yes	Yes	Yes	Yes	Yes
Analysis by intention-to-treat status	No	No	No	No	No	No	No	Can't tell

### Guidelines Characteristics

The gray literature searches identified 12 guidelines published since 2014 (Table 5). Seven were published in the USA (Higher Education Mental Health Alliance, 2014; Survivors of Suicide Loss Task Force, 2015; California Mental Health Services Authority, 2016; New York City Fire Department, 2016; Active Minds, 2017; National Center for School Crisis and Bereavement, 2017; American Foundation for Suicide Prevention, 2018), three in Australia (Headspace School Support, 2015; Department for Education and Child Development, 2016; Australian Institute for Suicide Research and Prevention, 2017), one in Canada (Centre for Suicide Prevention, 2019), and one in the UK (Public Health England, 2016). Seven guidelines were targeted at schools (Headspace School Support, 2015; Department for Education and Child Development, 2016; Active Minds, 2017; National Center for School Crisis and Bereavement, 2017; American Foundation for Suicide Prevention, 2018; Centre for Suicide Prevention, 2019) or colleges/universities (Higher Education Mental Health Alliance, 2014). Four guidelines aimed to assist (community) organizations and/or professionals helping all those bereaved by suicide (Survivors of Suicide Loss Task Force, 2015; California Mental Health Services Authority, 2016; Public Health England, 2016; Australian Institute for Suicide Research and Prevention, 2017), and one guideline specifically focused on a workplace environment (firefighters) (New York City Fire Department, 2016). All guidelines described their objectives. Seven guidelines described the methods of their development (Higher Education Mental Health Alliance, 2014; Headspace School Support, 2015; Survivors of Suicide Loss Task Force, 2015; California Mental Health Services Authority, 2016; New York City Fire Department, 2016; Australian Institute for Suicide Research and Prevention, 2017; American Foundation for Suicide Prevention, 2018), and the users were involved in the development of eight guidelines (Higher Education Mental Health Alliance, 2014; Headspace School Support, 2015; Survivors of Suicide Loss Task Force, 2015; California Mental Health Services Authority, 2016; New York City Fire Department, 2016; Public Health England, 2016; Australian Institute for Suicide Research and Prevention, 2017; American Foundation for Suicide Prevention, 2018). The evidence-base, described in ten guidelines mostly comprised a combination of references to literature and an expert advisory group or a consensus procedure, such as a Delphi study (Higher Education Mental Health Alliance, 2014; Headspace School Support, 2015; Survivors of Suicide Loss Task Force, 2015; California Mental Health Services Authority, 2016; New York City Fire Department, 2016; Public Health England, 2016; Active Minds, 2017; Australian Institute for Suicide Research and Prevention, 2017; American Foundation for Suicide Prevention, 2018; Centre for Suicide Prevention, 2019). Three guidelines described their theoretical model of postvention, i.e., a public health model (Survivors of Suicide Loss Task Force, 2015; Public Health England, 2016; Australian Institute for Suicide Research and Prevention, 2017). While three guidelines (California Mental Health Services Authority, 2016; Department for Education and Child Development, 2016; Active Minds, 2017) provided key recommendations, six provided sample material such as templates of letters (Higher Education Mental Health Alliance, 2014; California Mental Health Services Authority, 2016; Department for Education and Child Development, 2016; Active Minds, 2017; National Center for School Crisis and Bereavement, 2017; American Foundation for Suicide Prevention, 2018).

**Table 5:** Summary of guidelines^a^ (*n* = 12).

**TitleAuthorCountry, Year**	**Target users**	**Target population**	**Objectives described**	**Development methods described**	**Target users included in development**	**Evidence-base described**	**Theory of postvention described**	**Key recommendations included**	**Sample material included**	**Source**
*After a campus suicide: A postvention guide for student-led responses*Active MindsUSA, 2017	Students leading a campus-wide response to suicide	Schools after a student suicide	Yes	No	Unknown	Yes (Literature)	No	Yes	Yes (Social media postings)	https://www.activeminds.org/programs/after-a-campus-suicide-postvention-guide/
*After a suicide: A Toolkit for schools, 2*nd *Ed*.American Foundation for Suicide Prevention, Suicide Prevention Resource Center, Education Development CenterUSA, 2018	School administrators, staff, parents, communities	Schools after a suicide in the school community	Yes	Yes	Yes	Yes (Consensus procedure and Literature; Ref to NSSP)	No	No	Yes (Various letters, meeting agendas)	http://www.sprc.org/sites/default/files/resource-program/AfteraSuicideToolkitfor Schools.pdf
*After rural suicide: A guide for coordinated community postvention response*California Mental Health Services AuthorityUSA, 2016	Local public health, law enforcement, suicide prevention coalitions	Local community after a suicide	Yes	Yes	Yes	Yes (Consensus procedure and Literature; Ref to NSSP)	No	Yes	Yes (Various checklist, letters, flyers)	https://www.cibhs.org/sites/main/files/file-attachments/after_rural_suicide_ guide_2016_rev.docx
*A suicide prevention toolkit: After a student suicide*Centre for Suicide PreventionCanada, 2019 (update from 2016)	Schools	Schools after a student suicide	Yes	No	Unknown	Yes (Literature)	No	No	No (link to AFSP 2018 guidelines, above)	https://www.suicideinfo.ca/wp-content/uploads/2016/03/After_a_student_suicide_web.pdf
*Guidelines for schools responding to a death by suicide*National Center for School Crisis and Bereavement, USC Suzanne Dworak-Peck School of Social WorkUSA, 2017	School administrators, teachers, and crisis team members	Schools after a suicide in the school community	Yes	No	Unknown	No	No	No	Yes (Various letters via link)	https://www.schoolcrisiscenter.org/resources/guide-responding-suicide/
*Guidelines for suicide postvention in fire service (Standard Operating Procedure)*New York City Fire DepartmentUSA, 2016	Firefighters peer support	Firefighters affected by suicide	Yes	Yes	Yes	Yes (Expert and Focus Groups consensus study)	No	No	No	https://www.tandfonline.com/doi/pdf/10.1080/07481187.2015.1077357?needAccess=true
*Postvention: A Guide for response to suicide on college campuses*Higher Education Mental Health AllianceUSA, 2014	Colleges, universities	Campuses after a death by suicide	Yes	Yes	Yes	Yes (Literature, Expert review)	No	No	Yes (One sample letter)	https://adaa.org/sites/default/files/postvention_guide-suicide-college.pdf
*Postvention Australia guidelines: A resource for organizations and individuals providing services to people bereaved by suicide* Australian Institute for Suicide Research and Prevention, and Postvention AustraliaAustralia, 2017	Organizations and individuals providing services	People bereaved by suicide	Yes	Yes	Yes	Yes (Literature, Focus Groups, and expert review)	Yes	No	No	https://www.griffith.edu.au/__data/assets/pdf_file/0038/359696/Postvention_WEB.pdf
*Responding to grief, trauma, and distress after a suicide: U.S. national guidelines*Survivors of Suicide Loss Task Force, National Action Alliance for Suicide PreventionUSA, 2015	All professionals and peers wishing to help those impacted by suicide loss	People bereaved by suicide	Yes	Yes	Yes	Yes (literature, Taskforce, Expert Group review, Ref to NSSP)	Yes	No	No	https://theactionalliance.org/sites/default/files/inline-files/NationalGuidelines.pdf
*Responding to suicide in secondary schools: A Delphi Study*headspace School SupportAustralia, 2015	School communities	Schools after a student suicide	Yes	Yes	Yes	Yes (Literature and Delphi consensus study)	No	No	No	https://headspace.org.au/assets/School-Support/hSS-Delphi-Study-web.pdf
*Suicide postvention guidelines: A framework to assist staff in supporting their school communities in responding to suspected, attempted or suicide death*Department for Education and Child Development, Catholic Education SA Association of Independent Schools of SA, Child and Adolescent Mental Health Services SAAustralia, 2016	Schools	Suspected, attempted, and suicide death	Yes	No	Unknown	No	No	Yes	Yes (Various letters and scripts)	https://www.education.sa.gov.au/sites/g/files/net691/f/suicide-postvention-guidelines.pdf
*Support after a suicide: A guide to providing local services: A practice resource*Public Health England, and National Suicide Prevention AllianceUK, 2016	Commissioners, local health and wellbeing boards, others	People bereaved by suicide	Yes	No	Yes	Yes (Literature, Advisory group, Ref to national suicide prevention strategy)	Yes	No	No	https://assets.publishing.service.gov.uk/government/uploads/system/uploads/attachment_data/file/590838/support_after_a_suicide.pdf

a *Based on the criteria of the “Appraisal of Guidelines for Research and Evaluation II” (AGREE Next Steps Consortium, 2017)*.

### Research Question 1

Which suicide postvention service models have been shown to be effective to reduce distress in family, friends and communities following a suicide?

#### Research Studies (N = 8)

Research studies have found little evidence of effectiveness of interventions. Only five studies reported a positive outcome of their intervention (Visser et al., 2014; Kramer et al., 2015; Supiano et al., 2017; Cha et al., 2018; Zisook et al., 2018). A school-based intervention (Cha et al., 2018) and two intensive grief psychotherapy programs (Supiano et al., 2017; Zisook et al., 2018) found improvement in grief scores, including complicated grief (Zisook et al., 2018). School-based intervention (Cha et al., 2018) and an online support forum (Kramer et al., 2015) reported an improvement in mental health scores. A community-based crisis intervention program and an intensive grief therapy program reported decreases in suicidality (Visser et al., 2014; Zisook et al., 2018). In contrast, other measures in these studies, as well as the measures in the other studies (Wittouck et al., 2014; Peters et al., 2015; Scocco et al., 2019), including one RCT (Wittouck et al., 2014) yielded mixed results regarding grief, mental health or suicidality. Hence, while some evidence is emerging, this review found little evidence of effective models of postvention service delivery.

#### Guidelines (N = 12)

Most guidelines (*n* = 7) focused on postvention activities in school or college (Higher Education Mental Health Alliance, 2014; Headspace School Support, 2015; Department for Education and Child Development, 2016; Active Minds, 2017; National Center for School Crisis and Bereavement, 2017; American Foundation for Suicide Prevention, 2018; Centre for Suicide Prevention, 2019). School postvention guidelines can play an important role in service provision considering that students bereaved by suicide might be at-risk of contagion. Furthermore, schools might be able to link at-risk students with counselors or mental health services. While most school guidelines were based on the literature, there were notable differences in their depth. Most guidelines covered the immediate period after death, including crisis response; while others focused more widely from preparations for potential suicides to ongoing support and monitoring. Also considered were the importance of (social) media and use of language. The most comprehensive examples would include “After a suicide: A toolkit for Schools” (American Foundation for Suicide Prevention, 2018), “Responding to suicide in secondary schools: A Delphi Study” (Headspace School Support, 2015), and “Suicide postvention guidelines” (Department for Education and Child Development, 2016).

The remaining five guidelines (Survivors of Suicide Loss Task Force, 2015; California Mental Health Services Authority, 2016; New York City Fire Department, 2016; Public Health England, 2016; Australian Institute for Suicide Research and Prevention, 2017) were diverse with four focusing on postvention in the wider community (such as “After rural suicide,” California Mental Health Services Authority, 2016), and one targeting a specific workplace (fire fighters) (New York City Fire Department, 2016). Three guidelines focused mainly on postvention service delivery: “Support after a suicide: A guide to providing local services” (Public Health England, 2016) provided a general overview; “Postvention Australia guidelines” (Australian Institute for Suicide Research and Prevention, 2017), concentrating on principles of postvention service provision for different organizations; The “US National Guidelines” (Survivors of Suicide Loss Task Force, 2015) provided an extensive literature review and a set of strategic directions. These three guidelines adopted a theoretical model of postvention service delivery, based on a public health approach (Survivors of Suicide Loss Task Force, 2015; Public Health England, 2016; Australian Institute for Suicide Research and Prevention, 2017).

### Research Question 2

From the models identified in Question 1, what components of suicide postvention services have been determined to contribute to effectiveness?

Given the limited evidence found in the research studies included in this review, one must be cautious in identifying components that may have contributed to effectiveness of interventions. However, some potentially effective components are highlighted here. These can be understood in the context of a public health approach to postvention, as described in some of the guidelines (Survivors of Suicide Loss Task Force, 2015; Public Health England, 2016; Australian Institute for Suicide Research and Prevention, 2017).

#### Level of Support

In studies showing evidence of effectiveness there is a distinction between help offered to all individuals bereaved by suicide and help for those with higher levels of grief or mental health symptoms. Cha et al. (2018) distinguished between educational support for all bereaved students and a psychotherapeutic approach to those with high bereavement-related symptoms. Visser et al. (2014) distinguished between face-to-face early outreach to all suicide bereaved individuals and referral to treatment as needed, and Supiano et al. (2017) and Zisook et al. (2018) offered manualized intensive grief therapy to individuals with high levels of grief symptoms.

#### Peer Support and Involvement

Qualitative data reported by participants in online discussion forums (Kramer et al., 2015) and a community-based program (Peters et al., 2015) pointed to the importance of finding recognition of one's grief, sharing experiences, and providing and receiving peer-support. Also, the positive effects found in a residential treatment program might be attributed, at least partly, to the social support experienced during the residential stay (Scocco et al., 2019).

#### Grief Focus

Another common factor of the effective interventions seems a focus on the grief of the individuals bereaved by suicide. While this seems obvious, three studies did not measure grief in participants (Visser et al., 2014; Peters et al., 2015; Scocco et al., 2019).

Correspondingly, three guidelines described their theoretical model of postvention (Survivors of Suicide Loss Task Force, 2015; Public Health England, 2016; Australian Institute for Suicide Research and Prevention, 2017), i.e., public health models taking into consideration the notion of a continuum of survivorship (i.e., needs of help of the bereaved individuals may differ depending on the experienced level of impact of the suicide). The US national postvention guidelines (Survivors of Suicide Loss Task Force, 2015) were based on the framework used by the US National Strategy for Suicide Prevention (U.S. Department of Health and Human Services, 2012), comprising universal, selective, and indicated strategies, and research and evaluation. The UK Support after a Suicide guidelines (Public Health England, 2016) also referred to the public health model developed by the UK national suicide prevention strategy. It differentiates four levels of help that are offered to all the bereaved by suicide, to those in need of social support, to those who are strongly affected, and those who need specialized psychotherapy. Also the Postvention Australia guidelines (Australian Institute for Suicide Research and Prevention, 2017) adopted a similar four-level model of service delivery. Table 6 summarizes these three models (Survivors of Suicide Loss Task Force, 2015; Public Health England, 2016; Australian Institute for Suicide Research and Prevention, 2017). It is understood that the number of bereaved people is largest in the lowest level (universal interventions) and smallest in the top level (indicated interventions). Together these guidelines also stress the need for training of service providers and rigorous surveillance, research and evaluation of interventions and service delivery.

**Table 6:** Postvention service delivery according to level of impact of suicide.

**Level of postventive interventions according to actions recommended in guidelines**	**Responding to grief, trauma, and distress after a suicide: U.S. national guidelines Survivors of Suicide Loss Task Force (2015)**	**Support after a suicide: A guide to providing local services: A practice resourcePublic Health England (2016)^a^**	**Postvention Australia guidelines: A resource for organizations and individuals providing services to people bereaved by suicide Australian Institute for Suicide Research and Prevention (2017)**	
Indicated interventions for people with mental health problems and disordered grief	Indicated interventions: evidence-based treatments, communication between service providers	In-depth therapy, one-to-one psychological help provided by qualified practitioners	Psychotherapy	Surveillance, research and evaluation
Selective intervention for people with severe grief reactions, strongly impacted	Implementation of guidelines, training of service providers, availability of services	Therapeutic/psychoeducational, one-to-one support, and facilitated “closed” groups provided by qualified practitioners and trained facilitators	Counseling	
Selective interventions for people with moderate grief reactions, mildly impacted		Self-help, peer support, “open” groups, and remembrance events organized by voluntary and peer groups	Support services, support groups, self-help groups, helplines, community and educational support	
Universal interventions for people with low levels of grief, little impact of suicide	Information and awareness of postvention in general public, professionals and organizations	Information on grief and bereavement by suicide and signposting to sources of support by local or national organizations	Information including leaflets, books, booklets, factsheets, posters and online information	

a *Two resources “Support after a suicide: Developing and delivering local bereavement support services” (http://www.nspa.org.uk/wp-content/uploads/2017/01/NSPA-postvention-framework-20.10.16.pdf) and “Support after a suicide: Evaluating local bereavement support services” (http://www.nspa.org.uk/wp-content/uploads/2017/01/NSPA-postvention-evaluation-24.10.16.pdf) complement the guideline*.

Of note, the guidelines that did not refer to a theoretical model of postvention, such as the school-oriented guidelines (Higher Education Mental Health Alliance, 2014; Headspace School Support, 2015; Department for Education and Child Development, 2016; Active Minds, 2017; National Center for School Crisis and Bereavement, 2017; American Foundation for Suicide Prevention, 2018; Centre for Suicide Prevention, 2019) seem mostly based on a crisis intervention model, including immediate response after a suicide, follow-up and referral of at-risk students, and links with external services. Such crisis intervention approaches can be incorporated in a larger public health approach.

## Discussion

### Discussion of Main Findings

This review was concerned with support for people bereaved by suicide and addressed the following two questions: (1) Which suicide postvention service models have been shown to be effective to reduce distress in family, friends and communities following a suicide? (2) From the models identified in question 1, what components of suicide postvention services have been determined to contribute to effectiveness?

A thorough search of the peer-reviewed and gray literature identified eight studies (Table 1) and twelve guidelines (Table 5) published since 2014. Overall, the studies included in this review involved diverse populations, settings, interventions, and measures, limiting the comparability of the findings. Most studies lacked a control group (Visser et al., 2014; Kramer et al., 2015; Peters et al., 2015; Supiano et al., 2017; Cha et al., 2018; Scocco et al., 2019), and overall study quality was weak. Still, five interventions resulted in positive outcomes regarding grief (Supiano et al., 2017; Cha et al., 2018; Zisook et al., 2018), mental health (Kramer et al., 2015; Cha et al., 2018), and suicidality (Visser et al., 2014; Zisook et al., 2018). The reviewed guidelines hold promise to inform and support suicide postvention services. However, except for three guidelines (Survivors of Suicide Loss Task Force, 2015; Public Health England, 2016; Australian Institute for Suicide Research and Prevention, 2017), all documents lacked a theoretical background, and no evaluations have been reported.

As this review was limited to publications since 2014 it is useful to consider additional evidence from earlier publications. A recent systematic review of effectiveness of controlled studies of interventions for people bereaved by suicide identified 11 studies published between 1984 and 2018 (Andriessen et al., 2019). That review found some evidence of effectiveness on grief outcomes of an 8-week support group program facilitated by a mental health professional and a trained volunteer (Farberow, 1992). A study comparing effects of a professionally led group psychotherapy and a social group program for widows bereaved through suicide found that grief symptoms reduced in the therapy group (Constantino and Bricker, 1996), although effects did not differ in a larger replication study (Constantino et al., 2001). A study comparing the effects of a death-related writing task intervention with a neutral writing task control condition yielded a significant reduction in grief levels in both groups, but more in the intervention group than in the control group (Kovac and Range, 2000).

Regarding psychosocial outcomes, the previous review (Andriessen et al., 2019) found that a 10-week psychologist-facilitated group therapy program for children reduced anxiety and depression but not posttraumatic stress of social adjustment at 12-weeks follow-up (Pfeffer et al., 2002). A psychoeducational component for parents may have contributed to the positive effects. A study of a series of three church-based support meetings following a suicide in the community found modest positive effects in the intervention group in terms of greater self-efficacy, social acceptance and job competency, up to 2 months after the intervention (Sandor et al., 1994). Together these studies suggest that social support in the community (Sandor et al., 1994), and a professionally led (with involvement of trained volunteers) support group or therapy group program for adults (Farberow, 1992) and for children (Pfeffer et al., 2002) might be helpful.

The components that might have contributed to positive effects of interventions in this review, were concerned with the different levels of grief or distress experienced by the bereaved, which is in line with public health models of postvention service delivery (Table 6). For example, informal, social support could be beneficial for all bereaved (Scocco et al., 2019). Those who are affected by suicide without symptoms of posttraumatic stress could benefit from an educational approach (Cha et al., 2018). Peer support, mutual recognition and sharing might be helpful for those mildly affected (Kramer et al., 2015), while those highly distressed and/or at-risk of disordered grief or ill mental health might benefit from specialized psychotherapy (Supiano et al., 2017; Zisook et al., 2018).

The recent systematic review identified additional potentially effective ingredients (Andriessen et al., 2019). The involvement of trained volunteers who serve as positive role models and peer supporters along mental health professional might contribute to effectiveness of support or therapy group effectiveness (Farberow, 1992). Pfeffer et al. (2002) suggested that psychoeducation of parents contributed to the effect of the intervention for bereaved children, as it enabled them to better support their children. Similarly, involvement of the wider community might contribute to the effectiveness of an intervention (Sandor et al., 1994). Also, it seems beneficial to deliver interventions over time (e.g., over 8–10 weeks) (Farberow, 1992; Pfeffer et al., 2002) or to use manuals or guidelines for the intervention (Kovac and Range, 2000; Pfeffer et al., 2002; Supiano et al., 2017; Zisook et al., 2018). Overall, grief specific interventions seem to yield stronger effect than interventions targeting other outcomes (Andriessen et al., 2019).

Most guidelines, especially school-based guidelines (Higher Education Mental Health Alliance, 2014; Headspace School Support, 2015; Department for Education and Child Development, 2016; Active Minds, 2017; National Center for School Crisis and Bereavement, 2017; American Foundation for Suicide Prevention, 2018; Centre for Suicide Prevention, 2019) are based on a crisis intervention approach. Callahan (1996) reported that an isolated school crisis intervention after a suicide might result in iatrogenic effects, such as increased distress and attempted suicide in students. Also, student suicide has a strong impact on school staff, who often feel ill-equipped to deal with it (Kõlves et al., 2017). Hence, it is recommended that school interventions are embedded in a whole-school approach, including suicide prevention and postvention training (Mackesy-Amiti et al., 1996; Robinson et al., 2016), and collaboration with specialized community mental health services (Rickwood et al., 2018).

Given that postvention is considered an important aspect of suicide prevention in Australia (Department of Health, 2017; Mental Health Commission of NSW, 2018) and internationally (World Health Organization, 2014), it seems logical to apply the same public health models to suicide postvention and prevention alike (Andriessen and Krysinska, 2012; World Health Organization, 2012). For example, the stepped-care model incorporated in the Fifth National Mental Health and Suicide Prevention Plan (Department of Health, 2017) fits well with the postvention models presented in the guidelines (Table 6) (Survivors of Suicide Loss Task Force, 2015; Public Health England, 2016; Australian Institute for Suicide Research and Prevention, 2017).

### Limitations

Regarding evidence from research, important gaps exist regarding effectiveness of interventions for different age and gender groups of the bereaved individuals. Only one study targeted young people (Cha et al., 2018), no study specifically focused on older adults, and men are underrepresented in almost all studies (Visser et al., 2014; Wittouck et al., 2014; Kramer et al., 2015; Peters et al., 2015; Supiano et al., 2017; Zisook et al., 2018; Scocco et al., 2019). No study addressed Indigenous populations.

Only one study evaluated the effectiveness of help offered through the Internet (Kramer et al., 2015). Given the omnipresence of the Internet and social media, more research in this area could identify potentially effective postvention interventions and their components. Also, only one study addressed early outreach (Visser et al., 2014) and the effect of this approach on suicide bereavement remains unclear. Further, while two psychotherapy studies reported positive findings (Supiano et al., 2017; Zisook et al., 2018), one psychotherapy RCT failed to find evidence of effectiveness in comparison to the control group (Wittouck et al., 2014).

Due to lack of control groups, little is known of effectiveness of potentially effective components, such as psychoeducation, finding recognition of one's grief, sharing experiences and receiving/providing peer support. While suicide bereavement support groups are widely available, no study in this review examined their effectiveness. Moreover, many services for people bereaved by suicide have been founded by the bereaved themselves. However, it is unknown if these services are now more accessible to bereaved individuals than statutory services. The studies and guidelines included in this review involved both types of services. There may also be differences across countries. A future study might shed light on similarities or differences in service delivery according to the type of organization.

All the reviewed guidelines have great potential to inform, support, and complement existing services. Nevertheless, there is a need to evaluate their implementation and effectiveness. Inclusion of target groups and service providers in guideline development should ensure the feasibility and acceptability of guidelines. Adopting a theoretical (e.g., public health) model of postvention, training of service providers, and scientific evaluation of guidelines should maximize their impact and efficacy.

## Conclusions

This review found limited evidence of effectiveness of postvention interventions and service delivery, mainly due to a relative shortage of research, particularly high-quality research involving control groups. Systematic searches of the peer-reviewed and gray literature identified eight research studies reporting on a variety of individual and group interventions, and 12 guidelines targeted at schools or the wider community. While this review identified serious gaps in the knowledge, it also identified several potentially effective components of postvention, such as involvement of trained volunteers/peers, and focusing the interventions on the grief.

Adopting a public health framework for postvention service delivery offers the opportunity to tailor support to bereaved individuals according to the impact of suicide on their lives. This can range from information and awareness raising targeting all people bereaved by suicide to specialized psychotherapy for those bereaved people who experience high levels of grief and symptoms of poor mental health. Such a framework might also align postvention with suicide prevention and mental health programs.

## Author Contributions

KA and KKr searched the peer reviewed literature and extracted the data. KA, KKr, and NR conducted the quality assessment. KKõ and KA searched the gray literature and guidelines, and KA and KKr extracted the data. KA drafted the manuscript. All authors contributed to the design of the study, revisions of the draft, and agreed with the final draft.

### Conflict of Interest

The authors declare that the research was conducted in the absence of any commercial or financial relationships that could be construed as a potential conflict of interest.
