# Supplementary material for: Suicide Postvention Service Models and Guidelines 2014–2019: A Systematic Review
Source: Front Psychol. 2019 Nov 29;10:2677. doi: 10.3389/fpsyg.2019.02677 (PMC6896901; doi:10.3389/fpsyg.2019.02677)
Supplement: Supplementary file 1 [file Table_1.docx]

Suicide postvention service models and guidelines 2014-2019: A systematic review

# Supplementary material

## Table 1: NHMRC Levels of evidence

| **Level of evidence** | **Study design** |
| --- | --- |
| I | A systematic review of Level II studies. |
| II | A randomized controlled trial. |
| III-1 | A pseudo-randomized controlled trial (i.e., alternate allocation or some other method). |
| III-2 | A comparative study with concurrent controls (i.e., non-randomized experimental trials, cohort studies, case-control studies, interrupted time series studies with a control group). |
| III-3 | A comparative study without concurrent controls (i.e., historical control study, two or more single arm studies, interrupted time series studies without a parallel control group). |
| IV | Case series with either post-test or pre-test/post-test outcomes. |

## Table 2: NHMRC matrix to summarize the evidence base

| **Component** | **A** | **B** | **C** | **D** |
| --- | --- | --- | --- | --- |
|  | Excellent | Good | Satisfactory | Poor |
| Evidence base ^A^ | Several level I or II studies with low risk of bias | One or two level II studies with low risk of bias or a systematic review or multiple level III studies with low risk of bias | Level III studies with low risk of bias, or level I or II studies with moderate risk of bias | Level IV studies, or level I to III studies with high risk of bias |
| Consistency ^B^ | All studies consistent | Most studies consistent and inconsistency may be explained | Some inconsistency reflecting genuine uncertainty around clinical questions | Evidence is inconsistent |
| Clinical impact | Very large | Substantial | Moderate | Slight or restricted |
| Generalizability | Population/s studied in body of evidence are the same as the target population in question | Population/s studied in the body of evidence are similar to the target population in question | Population/s studied in body of evidence differ to target population in question but it is clinically sensible to apply this evidence to target population | Population/s studied in body of evidence differ to target population and hard to judge whether it is sensible to generalize to target population |
| Applicability | Directly applicable to Australian context | Applicable to Australian context with few caveats | Probably applicable to Australian context with some caveats | Not applicable to Australian context |

# ^A^ Level of evidence determined from the NHMRC evidence hierarchy as in Table 1 (above).

# ^B^ If there is only one study, rank this component as ‘not applicable’. National Health and Medical Research Council (2009) NHMRC levels of evidence and grades for recommendations for guideline developers. Canberra: National Health and Medical Research Council.
